# Oxidation of P700 Induces Alternative Electron Flow in Photosystem I in Wheat Leaves

**DOI:** 10.3390/plants8060152

**Published:** 2019-06-05

**Authors:** Kanae Kadota, Riu Furutani, Amane Makino, Yuji Suzuki, Shinya Wada, Chikahiro Miyake

**Affiliations:** 1 Department of Biological and Environmental Sciences, Faculty of Agriculture, Kobe University, 1-1 Rokkodai, Nada, Kobe 657-8501, Japan; k.kadota.0703@gmail.com (K.K.); 1626435a@stu.kobe-u.ac.jp (R.F.); swada@penguin.kobe-u.ac.jp (S.W.); 2 Graduate School of Agricultural Science, Tohoku University, Aramaki-Aoba 468-1, Aoba-ku, Sendai 980-8572, Japan; amanemakino@tohoku.ac.jp; 3 Faculty of Agriculture, Iwate University, 3-18-8 Ueda, Morioka, Iwate 020-8550, Japan; ysuzuki@iwate-u.ac.jp

**Keywords:** charge recombination, cyclic electron flow, ferredoxin, photosystem I, P700, reactive oxygen species

## Abstract

Oxygen (O_2_)-evolving photosynthetic organisms oxidize the reaction center chlorophyll, P700, in photosystem I (PSI) to suppress the production of reactive oxygen species. The oxidation of P700 is accompanied by alternative electron flow in PSI (AEF-I), which is not required for photosynthetic linear electron flow (LEF). To characterize AEF-I, we compared the redox reactions of P700 and ferredoxin (Fd) during the induction of carbon dioxide (CO_2_) assimilation in wheat leaves, using dark-interval relaxation kinetics analysis. Switching on an actinic light (1000 μmol photons m^−2^ s^−1^) at ambient CO_2_ partial pressure of 40 Pa and ambient O_2_ partial pressure of 21 kPa gradually oxidized P700 (P700^+^) and enhanced the reduction rate of P700^+^ (vP700) and oxidation rate of reduced Fd (vFd). The vFd showed a positive linear relationship with an apparent photosynthetic quantum yield of PSII (Y[II]) originating at point zero; the redox turnover of Fd is regulated by LEF via CO_2_ assimilation and photorespiration. The vP700 also showed a positive linear relationship with Y(II), but the intercept was positive, not zero. That is, the electron flux in PSI included the electron flux in AEF-I in addition to that in LEF. This indicates that the oxidation of P700 induces AEF-I. We propose a possible mechanism underlying AEF-I and its physiological role in the mitigation of oxidative damage.

## 1. Introduction

Suppression of the carbon dioxide (CO_2_) assimilation efficiency of photosynthesis under conditions of high light intensity, low/high temperature, or drought decreases the regeneration efficiency of the electron acceptor (NADP^+^) in photosystem I (PSI) and increases the risk of the accumulation of electrons in PSI of thylakoid membranes [1,2,3,4,5]. The reaction center chlorophyll (Chl), P700, is the electron source in PSI and drives the electron transport reaction from plastocyanin (PC) to NADP^+^ through ferredoxin (Fd). In the sunflower (*Helianthus annuus*), exposure of intact leaves to repetitive short-pulse high light intensity (rSP-illumination treatment) in the dark inactivates the PSI electron transport reaction [6]. Short-pulse illumination causes the accumulation of electrons on the acceptor side of PSI, which stimulates the production of reactive oxygen species (ROS) such as superoxide radicals and singlet oxygen [6,7,8]. By contrast, the rSP-illumination treatment under steady-state actinic light (AL) oxidizes P700, but does not lead to the accumulation of electrons or inactivation of PSI. These data suggest that the accumulation of electrons on the acceptor side of PSI increases the risk of ROS production, which inactivates PSI and CO_2_ assimilation [9,10,11].

Photosynthetic organisms employ diverse molecular mechanisms for the oxidation of P700 in PSI. The stimulation of photo-excited P700 (P700*) oxidation and/or suppression of the reduction of oxidized P700 (P700^+^) during the turnover of P700 in PSI accelerates P700 oxidation. Additionally, flavodiiron (FLV)-dependent electron flow and photorespiration stimulate P700* oxidation to maintain P700 in an oxidized state [10]. Furthermore, photosynthetic linear electron flow (LEF) during CO_2_ assimilation and photorespiration induces the accumulation of protons (H^+^) in the lumen of the thylakoid membrane. The acidification of the luminal side suppresses cytochrome *b*_6_/*f*-complex-catalyzed plastoquinol oxidation, which contributes to the oxidation of P700 [4,12]. The accumulation of H^+^ is controlled by ATP synthase-mediated utilization of ADP and Pi in the thylakoid membrane during CO_2_ assimilation and photorespiration [10,13,14,15,16]. These molecular mechanisms that lead to the oxidation of P700 are collectively referred to as the P700 oxidation system [10].

Enhanced electron fluxes in both FLV-dependent electron flow and photorespiration contribute to the oxidation of P700 in PSI, as described above. On the other hand, we have observed that P700 oxidation is accompanied by excess electron flow in PSI, which is not driven by LEF; this excess electron flow is referred to as the alternative electron flow in PSI (AEF-I) [17,18]. In this study, we performed a molecular characterization of AEF-I in wheat (*Triticum aestivum*) leaves using the DUAL/KLAS-NIR spectrophotometer (Walz, Germany), a novel spectrophotometer that specifically detects P700^+^, oxidized PC (PC^+^), and reduced Fd (Fd^−^) [19,20]. During the induction of AEF-I, we evaluated the relationship among the redox states of P700, PC, and Fd. Furthermore, we studied the relationship among the reduction rates of both P700^+^ and PC^+^ and oxidation rate of Fd^−^ using dark-interval relaxation kinetics (DIRK) analysis [1,21]. These analyses helped us understand the mechanism that regulates the activation of AEF-I and the relationship between P700 oxidation and AEF-I activity. Furthermore, we investigated the physiological function of P700 oxidation in photosynthetic organisms. The redox turnover rate of P700 was much higher than that of Fd and showed an absolute dependence on the electron flux in the LEF in wheat leaves. In other words, electron flux in AEF-I was induced by P700 oxidation and functioned within PSI. On the other hand, P700 oxidation contributed to the oxidation of Fd^−^, thus revealing a novel function of P700 oxidation. We propose a molecular mechanism for AEF-I and its physiological function in the mitigation of oxidative stress by suppressing the production of ROS in PSI.

## 2. Results

### 2.1. Effect of Ambient CO_2_ Partial Pressure on the Reduction Rates of PC^+^ and P700^+^ and Oxidation Rate of Fd^−^

To modulate the electron flux in the LEF in wheat leaves, we manipulated the partial pressure of ambient CO_2_ (pCO_2_). Generally, a reduction in pCO_2_ decreases the electron flux in the LEF, thus oxidizing the reaction center Chl, P700, in PSI [17,18]. This situation is advantageous, as it enhances our understanding of the effects of P700 oxidation on the relationship between P700^+^ reduction rate and Fd^-^ oxidation rate.

**Figure 1:**
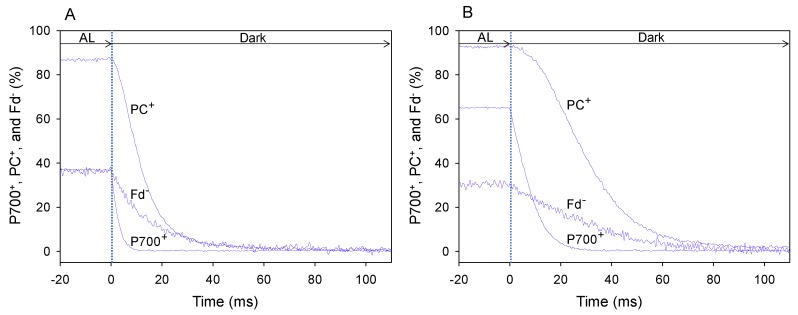
DIRK analysis of the decay of oxidized P700 (P700^+^), PC (PC^+^), and reduced Fd (Fd^−^) in wheat leaves after turning off actinic light (AL) illumination. Changes in the redox state of P700, PC, and Fd were monitored using a Dual/KLAS-NIR spectrophotometer [19,20]. To determine the reduction rates of P700^+^ and PC^+^ and oxidation rate of Fd^−^ in wheat leaves under the illuminated condition, AL was transiently turned off at time zero for 400 ms. The initial slope of the decrease in P700^+^, PC^+^, and Fd^−^ at time zero indicated the reduction rates of P700^+^ and PC^+^ and oxidation rate of Fd^−^. The initial slope changes of P700^+^, PC^+^, and Fd^−^ were characterized by averaging 70 sets of measurements at 25 °C leaf temperature, 21 kPa pO_2_, and 1000 mol photons m^−2^ s^−1^ light intensity, with either 40 Pa pCO_2_ (**A**) or 5 Pa pCO_2_ (**B**) for 110 ms after the AL was turned off. These data were obtained at a steady state, which was confirmed by the achievement of stable Y(II).

We set pCO_2_ at 40, 20, and 5 Pa in a stepwise manner, with a photon flux density of 1000 μmol photons m^−2^ s^−1^ and an ambient oxygen partial pressure (pO_2_) of 21 kPa, in wheat leaves. The typical kinetics of the reduction of PC^+^ and P700^+^ and oxidation of Fd^−^ at the highest and lowest pCO_2_ (40 and 5 Pa, respectively) were obtained using DIRK analysis after the electron flux in LEF reached a steady state, which was determined based on the stable values of quantum yield of PSII (Y[II]) (Figure 1) [21,22].

The steady-state level of P700^+^ under AL illumination at 40 Pa pCO_2_ (Figure 1A) was lower than that at 5 Pa pCO_2_ (Figure 1B). Additionally, the steady-state level of PC^+^ at 40 Pa pCO_2_ (Figure 1A) was slightly lower than that at 5 Pa pCO_2_ (Figure 1B), whereas the steady-state level of Fd^−^ at 40 Pa pCO_2_ was higher than that at 5 Pa pCO_2_ (Figure 1B).

In the DIRK analysis, AL illumination was transiently turned off for 400 ms. From the decay of oxidized P700 (P700^+^), oxidized PC (PC^+^) and reduced Fd (Fd^−^), reduction rates of P700^+^ and PC^+^ and oxidation rate of Fd^−^ under AL illumination were estimated [21]. The rate of decrease in P700^+^ and PC^+^ and the reduction rate of P700^+^ and PC^+^ at 40 Pa pCO_2_ (Figure 1A) were higher than those at 5 Pa pCO_2_ (Figure 1B). Similarly, the rate of decrease in Fd^−^ and the oxidation rate of Fd^−^ at 40 Pa pCO_2_ (Figure 1A) was higher than that at 5 Pa pCO_2_ (Figure 1B). These results indicate that the turnover rates of redox reactions of P700^+^, PC^+^ and Fd^−^ at 40 Pa pCO_2_ are higher than those at 5 Pa pCO_2_.

**Figure 2:**
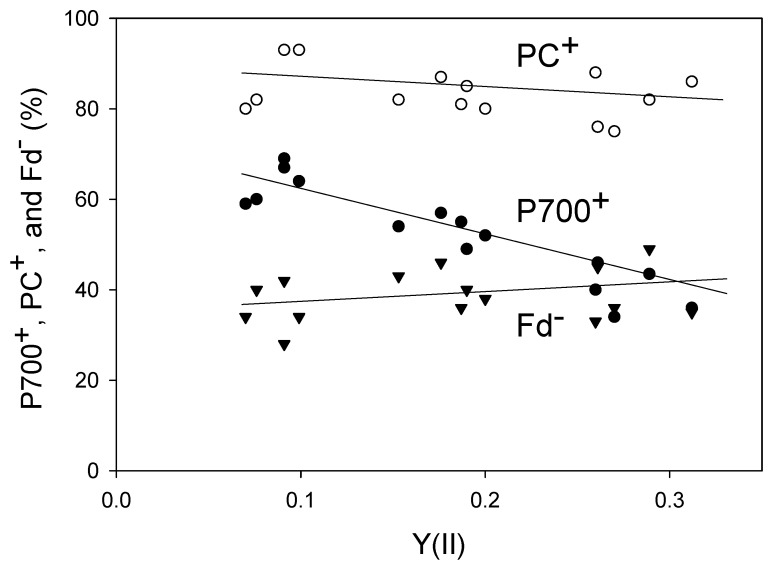
Relationships of P700^+^, PC^+^, and Fd^−^ with an apparent Y(II) in wheat leaves. Levels of P700^+^, PC^+^, Fd^−^ and Y(II) were measured simultaneously at 25 °C leaf temperature, 21 kPa pO_2_, and 1000 mol photons m^−2^ s^−1^ light intensity by changing the pCO_2_ from 40 Pa to 20 Pa and then to 5 Pa in a stepwise manner. Data for P700^+^, PC^+^, and Fd^−^ were collected from five plants. A decrease in pCO_2_ lowered Y(II). The steady states for measurements at several pCO_2_ values were confirmed by the achievement of stable Y(II). Open circle, PC^+^; closed circle, P700^+^, reverse triangle, Fd^−^.

We plotted the steady-state levels of P700^+^, PC^+^, and Fd^−^ against Y(II) (Figure 2). Lowering the pCO_2_ decreased Y(II), indicating the suppression of LEF activity. With the reduction in Y(II), the level of PC^+^ increased (linear regression line: intercept = 90 ± 3 [*p* < 0.001]; slope = −29 ± 18 [*p* > 0.1]; coefficient of determination = 0.176 [*p* > 0.1]), whereas that of Fd^−^ decreased (linear regression line: intercept = 35 ± 4 [*p* < 0.001]; slope = 16 ± 18 [*p* > 0.1]; coefficient of determination = 0.066 [*p* > 0.1]). Like PC^+^, the level of P700^+^ also increased in response to the decrease in Y(II) (linear regression line: intercept = 74 ± 3 [*p* < 0.001]; slope = −120 ± 14 [*p* < 0.001]; coefficient of determination = 0.845 [*p* < 0.001]), which was consistent with the results of both Golding and Johnson [17] and Miyake et al. [18]. Furthermore, the level of non-photochemical quenching of Chl fluorescence (NPQ) increased with the decrease in Y(II) (Figure S1). The decrease in pCO_2_ induces lumen acidification of thylakoid membranes, which drives NPQ induction and P700 oxidation.

**Figure 3:**
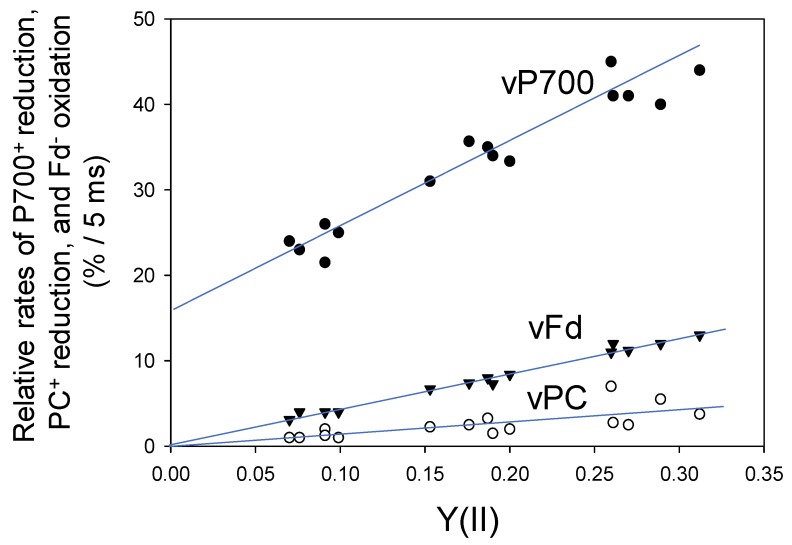
Relationships of vPC, vP700 and vFd with the apparent Y(II) in wheat leaves. Data points were expressed as relative changes (%) for 5 ms as initial changes from five plants (*n* = 5) after turning off of the AL, as described in Figure 1. A decrease in pCO_2_ lowered Y(II). Open circle, vPC; closed circle, vP700; reverse triangle, vFd.

Next, we evaluated the reduction rates of both P700^+^ and PC^+^ and the oxidation rate of Fd^−^ under steady-state conditions, using DIRK analysis (Figure 3). It is assumed that the initial rates of the redox changes in these components after the introduction of transient darkness reflect their rates of reduction (P700+ and PC+) or oxidation (Fd^−^) during illumination right before the darkness [21]. The relative reduction rates of P700^+^ (vP700) and PC^+^ (vPC) and relative oxidation rate of Fd^−^ (vFd), obtained from the initial 5 ms changes after turning off AL, were expressed as the percentage relative to the maximal level of each component. Initial changes of Fd^−^ oxidation was estimated under different pCO_2_ values, while simultaneously measuring Y(II), and plotted against Y(II). The relative rate of Fd^−^ oxidation clearly showed a positive linear relationship with Y(II) (linear regression line: intercept = 0.3 ± 0.3 [*p* > 0.05]; slope = 41.1 ± 1.3 [*p* < 0.001]; coefficient of determination = 0.987 [*p* < 0.001]). These results indicate that the turnover rate of Fd is determined by the photosynthetic LEF, which includes both CO_2_ assimilation and photorespiration; these results are consistent with those of a previous study examining *Arabidopsis thaliana* leaves [22].

Furthermore, the relative reduction rate of P700^+^ (vP700) showed a positive linear relationship with Y(II) (linear regression line: intercept = 16.6 ± 1.4 [*p* < 0.001]; slope = 92 ± 7 [*p* < 0.001]; coefficient of determination = 0.932 [*p* < 0.001]) (Figure 3). Unlike the relationship of vFd with Y(II), the intercept of the linear relationship of vP700 with Y(II) was not zero, indicating potential turnover of P700 in PSI at lower electron flux in LEF, as shown at zero of Y(II). This suggests that Fd-independent electron flow in PSI could function under suppressed photosynthesis.

The relative reduction rate of PC^+^ (vPC) also showed a positive linear relationship with Y(II) (linear regression line: intercept = −0.1 ± 0.8 [*p* > 0.1]; slope = 15 + 4 [*p* < 0.01]; coefficient of determination = 0.534 [*p* < 0.01]) (Figure 3). Like Fd, the redox reaction of PC was mainly determined by LEF.

### 2.2. AEF-I Functions in the Induction of Photosynthesis

**Figure 4:**
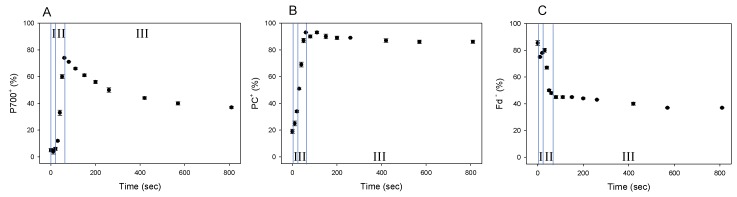
Responses of P700^+^, PC^+^, and Fd^−^ during the induction of photosynthesis in wheat leaves. Wheat leaves were illuminated using AL, which was turned on at 0 s. Levels of P700^+^ (**A**), PC^+^ (**B**), and Fd^−^ (**C**) were measured simultaneously at 25 °C leaf temperature, 40 Pa pCO_2_, 21 kPa pO_2_, and 1000 mol photons m^−2^ s^−1^ light intensity. Data represent mean ± standard deviation (SD; *n* = 5). I, phase I; II, phase II; III, phase III.

We showed that Fd-independent electron flow, which is referred to as AEF-I, functioned within PSI (Figure 3). Next, we tried to detect the electron flux in AEF-I during the induction of photosynthesis. When the AL was switched on, both P700 and PC were oxidized with a lag time in the first phase (phase I) (Figure 4A,B), reaching maximum oxidation (75% and 90%, respectively) at approximately 80 s in the second phase (phase II). In the third phase (phase III), the level of P700^+^ declined by approximately 40%, whereas that of PC^+^ remained largely unchanged. Illumination using AL rapidly increased the level of Fd^−^ to approximately 85% of that in phase I (Figure 4C). On the other hand, approximately 45% of Fd^−^ was oxidized at 80 s in phase II, and the level of Fd^−^ gradually decreased in phase III.

**Figure 5:**
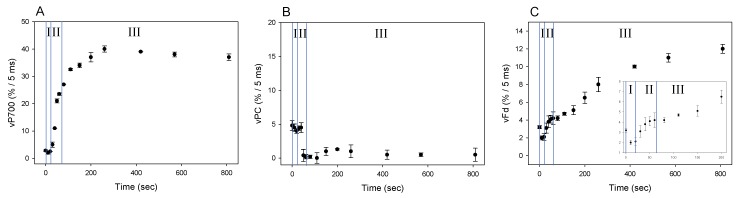
Responses of vP700, vPC, and vFd during the induction of photosynthesis in wheat leaves. Wheat leaves were illuminated using AL, which was turned on at 0 s. Values of vP700 (**A**), vPC (**B**), and vFd (**C**) were measured simultaneously at 25 °C leaf temperature, 40 Pa pCO_2_, 21 kPa pO_2_, and 1000 mol photons m^−2^ s^−1^ light intensity, as described in 4.3. The transient (400 ms) darkness for the DIRK analysis was introduced at the indicated time points. Data represent mean ± SD (*n* = 5). I, phase I; II, phase II; III, phase III.

We evaluated the changes in vP700, vPC, and vFd by DIRK analysis during the photosynthesis induction, where the redox changes of P700, PC and Fd showed three phases (phase I, II, and III: Figure 4). These facts suggested that each redox reaction rate would change in three phases (Figure 5). On AL illumination, vP700 increased to a maximum at approximately 250 seconds, with a lag time in phase I (Figure 5A). By contrast, vPC decreased considerably to levels approximating zero (Figure 5B), probably because of the rapid oxidation of PC^−^ by P700^+^, the level of which was in excess of 20%, as described above [21,23]. Change in vFd exhibited a complex pattern; vFd decreased rapidly from 3 to 2 (%/5 ms) over 20 s in phase I after the AL was switched on, then increased from 2 to 5 (%/5 ms) in phase II, and continued to increase in phase III, reaching a maximum of 12 (%/5 ms) at 800 s (Figure 5C). The pattern of increase in vFd resembled that observed in Y(II) during the induction of CO_2_ assimilation (Figure S2) [24]. In the lag phase of the induction of CO_2_ assimilation (phases I and II), the behavior of Y(II) did not match that of the net CO_2_ fixation rate, where an increased Y(II) showed that photorespiration would function. Photorespiration would start when the AL was switched on and would drive the photosynthetic LEF as a major electron sink in the lag phase (Figure 5C). A rapid start of photorespiration during the induction of CO_2_ assimilation has been demonstrated previously [25]. The increase in vFd in phase III was likely driven by the activated photosynthesis and photorespiration [24].

The behavior of the increase in vFd differed from that of vP700. Since vFd reflects the electron flux in LEF and vP700 can function independent on vFd (Figure 3), rapid increase of vP700 in phase II would show the activation of AEF-I. That is, during the induction of photosynthesis, AEF-I functions with the oxidation of P700 (Figure 4).

## 3. Discussion

**Figure 6:**
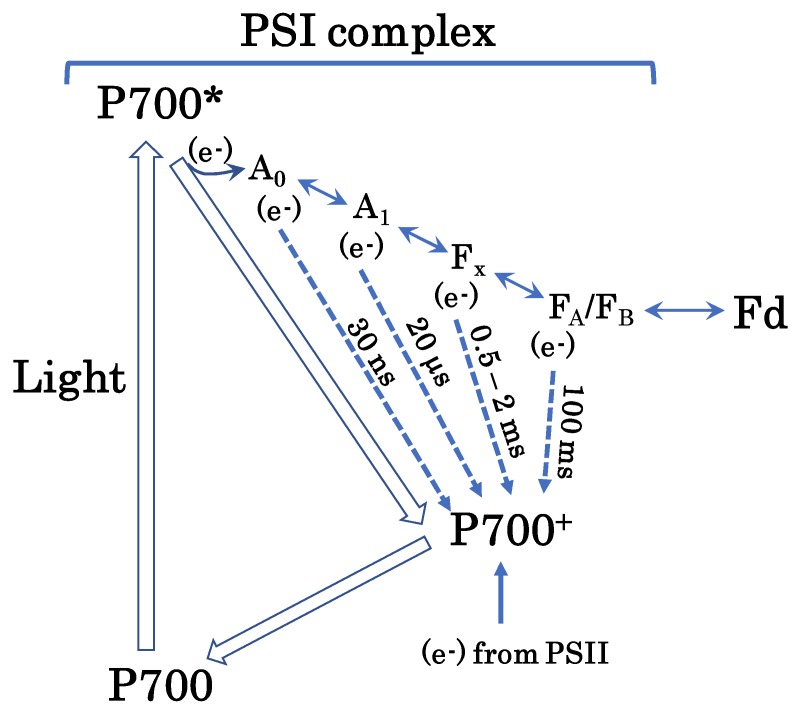
Hypothetical pathway of the AEF-I. Photo-excited P700 (P700*) donates an electron to the first electron carrier, A_0_, to produce P700^+^. Subsequently, P700^+^ accepts an electron from PC in PSII to regenerate P700. A_0_ donates the electron to the second electron carrier, A_1_. Thereafter, the electron flows to ferredoxin (Fd) through the third and fourth electron carriers, F_x_ and F_A_/F_B_, respectively. Empty arrows represent P700 turnover in the photo-oxidation reduction cycle. Solid arrows indicate electron flow. Dotted arrows indicate electron flow during charge recombination (please see text for further details). Charge recombination is one of the mechanisms of additional electron flow. This figure is a modification of original figures published previously [27,28,29,30,31,32,33,34,35].

We examined the effect of P700 oxidation on the redox state of Fd and activation of AEF-I. Switching on the AL to induce CO_2_ assimilation reduced Fd to approximately 90%; subsequently, the level of Fd^−^ decreased to 45%, while that of P700^+^ increased to approximately 80% (Figure 4 and Figure 5). These results indicate that the limitation of P700 turnover in PSI shifted from the acceptor side to the donor side [4,10,12,26]. The donor side limitation of P700 turnover was induced by enhanced LEF, as observed by the increase in vFd (phase II in Figure 4 and Figure 5), which contributed to P700 oxidation. The oxidation of P700 then induced AEF-I (Figure 5 and Figure 6).

We presume that charge recombination in PSI drives AEF-I (Figure 6). Once P700 is excited to P700*, it undergoes charge separation to produce P700^+^ and an electron [27,28,29,30]. The electrons released from P700* flow toward Fd via four electron-transfer cofactors: A_0A_/A_0B_, A_1A_/A_1B_, F_x_, and [F_A_/F_B_] [31,32,33,34,35]. The electrons accumulated in these cofactors then flow toward P700^+^; this phenomenon is referred to as a charge recombination. During this phenomenon, P700^+^ functions as an electron sink in the PSI of thylakoid membranes. The reduced electron-transfer cofactors A_0A_/A_0B_, A_1A_/A_1B_, F_x_, and [F_A_/F_B_] recombine with P700^+^ in approximately 30 ns, 20 μs, 0.5–2 ms, and 100 ms, respectively. In the present study, the half-time of the reduction of P700^+^ was in the same range as the recombination rate of P700^+^ with F_x_ or [F_A_/F_B_]. We propose that the AEF-I is driven by charge recombination of P700^+^ with the reduced form of F_x_ or [F_A_/F_B_] in PSI (Figure 6).

These electron-transfer cofactors in PSI donate electrons to O_2_ to produce superoxide radicals [6,7,8,36,37,38]. The O_2_ reduction rate constants of these cofactors are in the order of 10^6^ M^−1^ s^−1^ [32]. The apparent K_m_ for O_2_ in the photoreduction of O_2_ by PSI in thylakoid membranes is approximately 20 μM, which is approximately 1/10 of the O_2_ concentration in water equilibrated with atmospheric O_2_ (20.95%) [39]. This means that the photoreduction of O_2_ to superoxide radicals in thylakoid membranes is not limited by the availability of O_2_, but by that of electron donors on the reducing side of PSI. The accumulation of electrons in the electron-transfer cofactors, A_0A_/A_0B_, A_1A_/A_1B_, Fx, and [F_A_/F_B_], is dangerous for chloroplasts, as this enables the production of ROS. The recombination of these cofactors with P700^+^ inhibits the interaction between PSI and O_2_.

The charge recombination reactions between electron-transfer cofactors and P700^+^ are exergonic. The mid-point potentials of these cofactors are lower than that of P700^+^ [32]. Therefore, these recombination reactions dissipate energy as heat, which contributes to the alleviation of PSI photoinhibition under excess light energy [40].

It has been suggested for a long time that photosynthetic organisms operate Fd-dependent cyclic electron flows around PSI [41,42,43,44]. It is possible that Fd is an electron donor for Fd-quinone oxidoreductase (FQR) and NADH dehydrogenase (NDH). FQR requires protein cofactors, such as PGR5/PGRL1. Recently, a positive linear relationship between vFd and photosynthetic LEF rate during steady-state photosynthesis was demonstrated in *Arabidopsis* mutants deficient in PGR5/PGRL1 and NDH [22]. This also supports the hypothesis that Fd turnover is mainly determined by CO_2_ assimilation and photorespiration. Thus, both FQR- and NDH-dependent cyclic electron flows show negligible activity compared with photosynthetic LEF [45].

Furthermore, our conclusion that AEF-I is not driven by PGR5/PGRL1 or NDH is strongly supported by previous studies. Yamamoto et al. [46] clearly showed the recovery of AEF-I in PSI after the recovery of the P700 oxidation system. The *Arabidopsis* *PGR5*-deficient mutant did not maintain P700 in its oxidized state, nor did it show any AEF-I under fluctuating light [46]. A double mutant lacking *PGR5* and overexpressing FLV recovered AEF-I. The FLV stimulated the photosynthetic LEF and oxidized P700 in PSI, which induced the electron flux in AEF-I in both wild-type and PGR5-deficient *Arabidopsis* plants. This was further confirmed by the introduction of FLV in PGR5 deficient rice (*Oryza sativa*) plants [47]. Based on these studies, we conclude that AEF-I is not driven by PGR5/PGRL1 or NDH.

We propose a new physiological function of P700 oxidation. The oxidation of P700 suppresses the accumulation of electrons in the electron-transfer cofactors on the acceptor side of PSI by stimulating charge recombination in PSI, as described above. The oxidation of P700 also suppresses electron flow to Fd, as observed in the induction of Fd oxidation. The Fd^−^ donates electrons to O_2_ to produce a superoxide radical [48]. The superoxide radical disproportionates to O_2_ and H_2_O_2_, the latter of which is reduced by Fd^−^ to form the hydroxy radical, the most dangerous ROS [48]. Thus, P700 oxidation protects PSI by enhancing charge recombination, i.e., AEF-I, which suppresses the accumulation of electrons on the acceptor side of PSI, including Fd.

## 4. Materials and Methods

### 4.1. Plant Materials and Growth Conditions

The winter wheat cultivar ‘Norin 61′ was used in this study. Seeds were incubated on wet cotton at 4 °C for three days to promote synchronized germination. The moistened seeds were grown in a mixture of soil (Metro-Mix 350; Sun Gro Horticulture, Bellevue, WA, USA) and vermiculite (Konan, Osaka, Japan) in pots (7.5 cm length × 7.5 cm width × 6 cm depth). Plants were grown under standard air-equilibrated conditions in an environmentally controlled chamber set at 25 °C day/20 °C night temperature, 16 h light/10 h dark photoperiod, and 700–800 µmol photons m^−2^ s^−1^ light intensity. Plants were watered every other day with 0.1% Hyponex solution (N:P:K = 5:10:5; Hyponex, Osaka, Japan). Plants were grown for at least six weeks, and fully expanded mature leaves were harvested for further analysis.

### 4.2. Simultaneous Measurements of Chl Fluorescence and Gas Exchange

Chl fluorescence and gas exchange were measured simultaneously using Dual PAM-100 and GFS-3000 systems equipped with a 3010-DUAL gas exchange chamber (Heinz Walz, Effeltrich, Germany). The absolute pCO_2_ and pO_2_ were maintained at 40 Pa, and 21 kPa, respectively, and leaf temperature was maintained at 25 ± 1 °C. The relative humidity of the gas entering the leaf chamber was set at 60%. Chl fluorescence parameters were calculated as follows [1]:

NPQ = (Fm/Fm’)−1(1)

Y(II) = (Fm’−Fs)/Fm’,(2)

where, Fm represents the maximum fluorescence yield; Fm’ represents the maximum variable fluorescence yield, and Fs represents the steady-state fluorescence yield.

### 4.3. Simultaneous Measurements of P700^+^, PC^+^, Fd^−^, and Chl Fluorescence

The redox states of P700, PC, Fd, and Chl fluorescence were simultaneously measured at 25 °C leaf temperature, 21 kPa pO_2_, and 1000 μmol photons m^−2^ s^−1^ light intensity by changing the pCO_2_ using a DUAL/KLAS-NIR spectrophotometer and GFS-3000 gas exchange system (Heinz Walz, Effeltrich, Germany) [19,20]. Leaf temperature was maintained at 25 ± 1 °C, and relative humidity of gas entering the leaf chamber was set at 60%. The maximum levels of P700 oxidation (100%) and PC oxidation (100%) were determined by illuminating saturated pulse light under far-red light, which reflected the maximum amounts of photo-oxidized P700 and photo-oxidized PC [19,20]. The maximum level of Fd reduction (100%) was determined by illuminating saturated pulse light on dark-adapted leaves under weak AL, which reflected the maximum amount of Fd^−^ [19,20]. The rapid changes in P700^+^, PC^+^, and Fd^−^ were determined by DIRK analysis [1,21,22].

## Supplementary Materials

The following are available online at https://www.mdpi.com/2223-7747/8/6/152/s1, Figure S1: Relationship of non-photochemical quenching of chlorophyll fluorescence (NPQ) with the apparent quantum yield of PSII (Y[II]) in wheat leaves, Figure S2: Response of the net CO_2_ assimilation rate and Y(II) to illumination by actinic light in wheat leaves.

## Abbreviations

Chl	chlorophyll
Fd	ferredoxin
LEF	photosynthetic linear electron flow
NPQ	non-photochemical quenching of chlorophyll fluorescence
PC	plastocyanin
pCO_2_	partial pressure of CO_2_
PSI	photosystem I
PSII	photosystem II
Y(II)	quantum yield of photochemical energy conversion in PSII

## References

[1] Baker N.R., Harbinson J., Kramer D.M. (2007). Determining the limitations and regulation of photosynthetic energy transduction in leaves. Plant Cell Environ..

[2] Gururani M.A., Venkatesh J., Tran L.S. (2015). Regulation of Photosynthesis during Abiotic Stress-Induced Photoinhibition. Mol. Plant.

[3] Li L., Aro E.M., Millar A.H. (2018). Mechanisms of Photodamage and Protein Turnover in Photoinhibition. Trends Plant Sci..

[4] Tikkanen M., Rantala S., Aro E.M. (2015). Electron flow from PSII to PSI under high light is controlled by PGR5 but not by PSBS. Front. Plant Sci..

[5] Tikkanen M., Rantala S., Grieco M., Aro E.M. (2017). Comparative analysis of mutant plants impaired in the main regulatory mechanisms of photosynthetic light reactions—From biophysical measurements to molecular mechanisms. Plant Physiol. Biochem..

[6] Sejima T., Takagi D., Fukayama H., Makino A., Miyake C. (2014). Repetitive short-pulse light mainly inactivates photosystem I in sunflower leaves. Plant Cell Physiol..

[7] Asada K. (2000). The water-water cycle as alternative photon and electron sinks. Philos. Trans. R. Soc. Lond. B Biol. Sci..

[8] Asada K., Kiso K., Yoshikawa K. (1974). Univalent reduction of molecular oxygen by spinach chloroplasts on illumination. J. Biol. Chem..

[9] Takagi D., Takumi S., Hashiguchi M., Sejima T., Miyake C. (2016). Superoxide and singlet oxygen produced within the thylakoid membranes both cause photosystem I photoinhibition. Plant Physiol..

[10] Shimakawa G., Miyake C. (2018). Oxidation of P700 ensures robust photosynthesis. Front. Plant Sci..

[11] Zivcak M., Brestic M., Kunderlikova K., Sytar O., Allakhverdiev S.I. (2015). Repetitive light pulse-induced photoinhibition of photosystem I severely affects CO_2_ assimilation and photoprotection in wheat leaves. Photosynth. Res..

[12] Tikhonov A.N. (2014). The cytochrome *b*_6_*f* complex at the crossroad of photosynthetic electron transport pathways. Plant Physiol. Biochem..

[13] Johnson M.P., Ruban A.V. (2014). Rethinking the existence of a steady-state ∆Ψ component of the proton motive force across plant thylakoid membranes. Photosynth. Res..

[14] Kohzuma K., Froehlich J.E., Davis G.A., Temple J.A., Minhas D., Dhingra A., Cruz J.A., Kramer D.M. (2017). The role of light-dark regulation of the chloroplast ATP synthase. Front. Plant Sci..

[15] Lyu H., Lazar D. (2017). Modeling the light-induced electric potential difference (∆Ψ), the pH difference (∆pH) and the proton motive force across the thylakoid membrane in C3 leaves. J. Theor. Biol..

[16] Takagi D., Hashiguchi M., Sejima T., Makino A., Miyake C. (2016). Photorespiration provides the chance of cyclic electron flow to operate for the redox-regulation of P700 in photosynthetic electron transport system of sunflower leaves. Photosynth. Res..

[17] Golding A.J., Johnson G.N. (2003). Down-regulation of linear and activation of cyclic electron transport during drought. Planta.

[18] Miyake C., Miyata M., Shinzaki Y., Tomizawa K. (2005). CO_2_ response of cyclic electron flow around PSI (CEF-PSI) in tobacco leaves—Relative electron fluxes through PSI and PSII determine the magnitude of non-photochemical quenching (NPQ) of Chl fluorescence. Plant Cell Physiol..

[19] Klughammer C., Schreiber U. (2016). Deconvolution of ferredoxin, plastocyanin, and P700 transmittance changes in intact leaves with a new type of kinetic LED array spectrophotometer. Photosynth. Res..

[20] Schreiber U., Klughammer C. (2016). Analysis of photosystem I donor and acceptor sides with a new type of online-deconvoluting kinetic LED-array spectrophotometer. Plant Cell Physiol..

[21] Sacksteder C.A., Kramer D.M. (2000). Dark-interval relaxation kinetics (DIRK) of absorbance changes as a quantitative probe of steady-state electron transfer. Photosynth. Res..

[22] Takagi D., Miyake C. (2018). Proton gradient regulation 5 supports linear electron flow to oxidize photosystem I. Physiol. Plant.

[23] Takagi D., Amako K., Hashiguchi M., Fukaki H., Ishizaki K., Goh T., Fukao Y., Sano R., Kurata T., Demura T. (2017). Chloroplastic ATP synthase builds up a proton motive force preventing production of reactive oxygen species in photosystem I. Plant J..

[24] Miyake C., Suzuki Y., Yamamoto H., Amako K., Makino A. (2012). O_2_-enhanced induction of photosynthesis in rice leaves: The Mehler-ascorbate peroxidase (MAP) pathway drives cyclic electron flow within PSII and cyclic electron flow around PSI. Soil Sci. Plant Nutr..

[25] Sejima T., Hanawa H., Shimakawa G., Takagi D., Suzuki Y., Fukayama H., Makino A., Miyake C. (2016). Post-illumination transient O_2_-uptake is driven by photorespiration in tobacco leaves. Physiol. Plant..

[26] Klughammer C., Schreiber U. (1994). An improved method, using saturating light pulses, for the determination of photosystem I quantum yield via P700^+^-absorbance changes at 830 nm. Planta.

[27] Brettel K., Leibl W. (2001). Electron transfer in photosystem I. Biochim. Biophys. Acta.

[28] Jordan P., Fromme P., Witt H.T., Klukas O., Saenger W., Krauss N. (2001). Three-dimensional structure of cyanobacterial photosystem I at 2.5 A resolution. Nature.

[29] Mazor Y., Borovikova A., Nelson N. (2015). The structure of plant photosystem I super-complex at 2.8 A resolution. Elife.

[30] Warren P.V., Golbeck J.H., Warden J.T. (1993). Charge recombination between P700+ and A1- occurs directly to the ground state of P700 in a photosystem I core devoid of FX, FB, and FA. Biochemistry.

[31] Brettel K. (1997). Electron transfer and arrangement of the redox cofactors in photosystem I. Biochim. Biophys. Acta.

[32] Charepanov D.A., Milanovsky G.E., Petrova A.A., Tikhonov A.N., Semenov A.Y. (2017). Electron transfer through the acceptor side of photosystem I: Interaction with exogenous acceptors and molecular oxygen. Biochemistry.

[33] Matsuoka T., Tanaka S., Ebina K. (2016). Reduced minimum model for the photosynthetic induction processes in photosystem I. J. Photochem. Photobiol. B.

[34] Shinkarev V.P., Vassiliev I.R., Golbeck J.H. (2000). A kinetic assessment of the sequence of electron transfer from F(X) to F(A) and further to F(B) in photosystem I: The value of the equilibrium constant between F(X) and F(A). Biophys. J..

[35] Shinkarev V.P., Zybailov B., Vassiliev I.R., Golbeck J.H. (2002). Modeling of the P700^+^ charge recombination kinetics with phylloquinone and plastoquinone-9 in the A1 site of photosystem I. Biophys. J..

[36] Asada K., Takahashi M., Kyle D.J., Osmond C.B., Arntzen C.J. (1987). Production and scavenging of active oxygen in photosynthesis. Photoinhibition.

[37] Hormann H., Neubauer C., Asada K., Schreiber U. (1993). Intact chloroplasts display pH 5 optimum of O_2_-reduction in the absence of methyl viologen: Indirect evidence for a regulatory role of superoxide protonation. Photosynth. Res..

[38] Takahashi M., Asada K. (1982). Dependence of oxygen affinity for Mehler reaction on photochemical activity of chloroplast thylakoids. Plant Cell Physiol..

[39] Miyake C., Yokota A. (2000). Determination of the rate of photoreduction of O_2_ in the water-water cycle in watermelon leaves and enhancement of the rate by limitation of photosynthesis. Plant Cell Physiol..

[40] Rutherford A.W., Osyczka A., Rappaport F. (2012). Back-reactions, short-circuits, leaks and other energy wasteful reactions in biological electron transfer: Redox tuning to survive life in O_2_. FEBS Lett..

[41] Munekage Y., Hojo M., Meurer J., Endo T., Tasaka M., Shikanai T. (2002). PGR5 is involved in cyclic electron flow around photosystem I and is essential for photoprotection in Arabidopsis. Cell.

[42] Shikanai T. (2016). Regulatory network of proton motive force: Contribution of cyclic electron transport around photosystem I. Photosynth Res..

[43] Yamamoto H., Shikanai T. (2019). PGR5-Dependent Cyclic Electron Flow Protects Photosystem I under Fluctuating Light at Donor and Acceptor Sides. Plant Physiol..

[44] Yamori W., Shikanai T. (2016). Physiological Functions of Cyclic Electron Transport Around Photosystem I in Sustaining Photosynthesis and Plant Growth. Annu. Rev. Plant Biol..

[45] Miyake C. (2010). Alternative electron flows (water-water cycle and cyclic electron flow around PSI) in photosynthesis: Molecular mechanisms and physiological functions. Plant Cell Physiol..

[46] Yamamoto H., Takahashi S., Badger M.R., Shikanai T. (2016). Artificial remodelling of alternative electron flow by flavodiiron proteins in Arabidopsis. Nat. Plants.

[47] Wada S., Yamamoto H., Suzuki Y., Yamori W., Shikanai T., Makino A. (2018). Flavodiiron Protein Substitutes for Cyclic Electron Flow without Competing CO_2_ Assimilation in Rice. Plant Physiol..

[48] Badger M.R. (1985). Photosynthetic oxygen exchange. Annu. Rev. Plant Biol..

