# Supplementary material for: Oxidation of P700 Induces Alternative Electron Flow in Photosystem I in Wheat Leaves
Source: Plants (Basel). 2019 Jun 5;8(6):152. doi: 10.3390/plants8060152 (PMC6631986; doi:10.3390/plants8060152)
Supplement: Supplementary file 1 [file plants-08-00152-s001.pdf]

Supplementary Figures

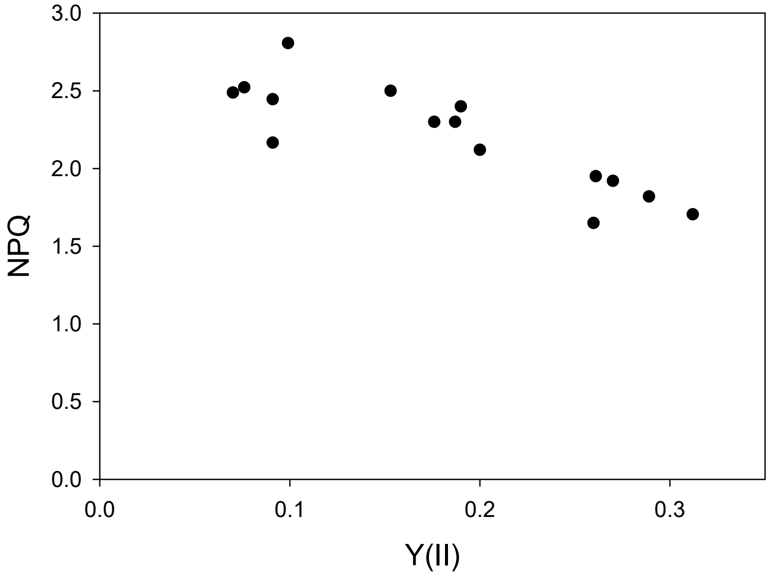

Figure S1. The relationship between the non-photochemical quenching of chlorophyll fluorescence (NPQ) and the apparent quantum yield of PSII (Y[II]) in wheat leaves. Both NPQ and Y(II) were measured simultaneously. Data points represent the averages of five plants. A reduction in pCO_2 lowered Y(II). Steady states for measurements at various pCO_2 were confirmed by the achievement of stable Y(II).

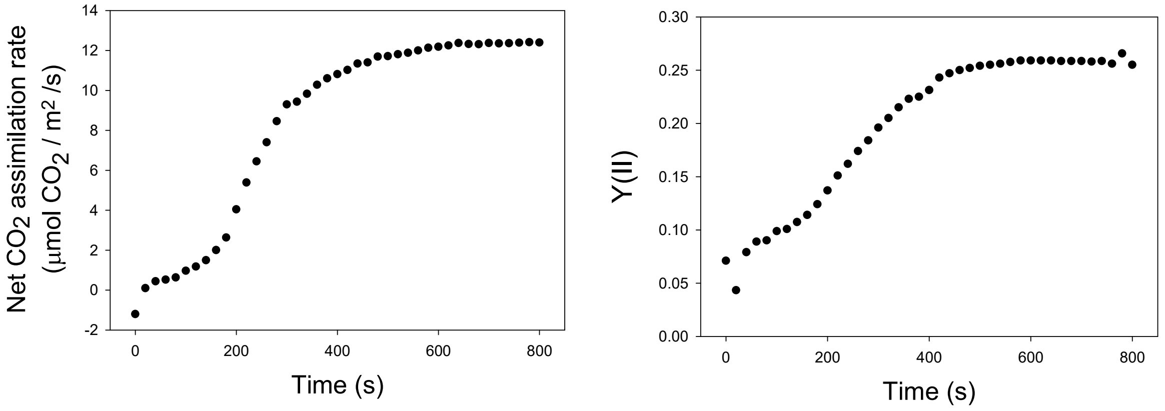

Figure S2. The responses of the net CO_2 assimilation rate and Y(II) to illumination by actinic light in wheat leaves. The net CO_2 assimilation rate (left panel) and Y(II) (right panel) were measured simultaneously at 25 °C leaf temperature, 40 Pa pCO_2, 21 kPa pO_2, and 1000 mol photons^{-1} m^{-2} s^{-1} light intensity. The actinic light was turned on at 0 s.
